# Identification of a substrate domain that determines system specificity in mycobacterial type VII secretion systems

**DOI:** 10.1038/srep42704

**Published:** 2017-02-16

**Authors:** Trang H. Phan, Roy Ummels, Wilbert Bitter, Edith N. G. Houben

**Affiliations:** 1 Section Molecular Microbiology, Amsterdam Institute of Molecules, Medicines & Systems, Vrije Universiteit Amsterdam, Amsterdam, The Netherlands; 2 Department of Medical Microbiology and Infection Control, VU University Medical Center, Amsterdam, The Netherlands

## Abstract

Type VII secretion (T7S) systems are specialized machineries used by mycobacterial pathogens to transport important virulence factors across their highly hydrophobic cell envelope. There are up to five mycobacterial T7S systems, named ESX-1 to ESX-5, at least three of which specifically secrete a different subset of substrates. The T7S substrates or substrate complexes are defined by the general secretion motif YxxxD/E. However this motif does not determine system specificity. Here, we show that the substrate domain recognized by the EspG chaperone is the determinant factor for this specificity. We first show that the introduction of point mutations into the EspG_1_-binding domain of the ESX-1 substrate pair PE35/PPE68_1 affects their secretion. Subsequently, we demonstrate that replacing this domain by the EspG_5_-binding domain of the ESX-5 substrate PPE18 resulted in EspG_5_ dependence and exclusive rerouting to the ESX-5 system. This rerouting of PE35/PPE68_1 to the ESX-5 system had a negative effect on the secretion of endogenous ESX-5 substrates.

*Mycobacterium tuberculosis*, the causative agent of tuberculosis, is an ancient, but still one of the deadliest human pathogens^1^. A highly distinctive feature of mycobacteria is the presence of a specific and highly impermeable outer membrane formed by mycolic acids and other unusual (glyco)lipids. To export virulence factors and other proteins across this special cell envelope, mycobacteria use specialized secretion systems known as type VII secretion (T7S) or ESX systems^2^. There are up to five paralogous *esx* loci present on the genome of pathogenic mycobacteria, named *esx-1* to *esx-5*^3^. The ESX-1 system, the first discovered T7S system in *M. tuberculosis*, plays a pivotal role in virulence and in completing the intracellular infection cycle of pathogenic mycobacteria by mediating phagosomal escape of the bacterium^4^. The ESX-5 system is the most-recently evolved system and is only present in slow-growing species of Mycobacteria, which include most pathogens, such as *M. tuberculosis, Mycobacterium leprae, Mycobacterium ulcerans* and *Mycobacterium marinum*^5^. We recently showed that ESX-5 is essential for *in vitro* growth of *M. marinum* and *Mycobacterium bovis* BCG and its functionality is linked to outer membrane permeabilization and nutrient uptake^6^.

The substrates of the different T7S systems can be divided in four subclasses, the Esx, PE, PPE and Esp proteins. The ESX-5 system is responsible for the secretion of most members of the large PE and PPE protein families^7^,^8^. Interestingly, the Esx proteins and at least some PE/PPE proteins are secreted as heterodimers^9^,^10^,^11^,^12^, forming a four-helix bundle. One of the partners in this heterodimer contains a general secretion motif, *i.e*. YxxxD/E, directly after the second α-helix^9^,^11^,^12^. However, this general secretion motif does not define system specificity^13^, as the C-terminal 15 amino acids of an ESX-5 substrate containing this motif could be replaced by the homologous sequence of an ESX-1 substrate, and *vice versa*, without changing system specificity^13^. It therefore remains unknown which signals define system specificity.

In other studies, we demonstrated that the cytosolic component EspG functions as a specific chaperone of PE/PPE proteins^11^,^14^. EspG_5_, the chaperone encoded by the *esx-5* locus, specifically interacts with PE/PPE proteins secreted via ESX-5, but not with an ESX-1 PE/PPE substrate pair^14^. The resolved structure of EspG_5_ in complex with PE25/PPE41 revealed that this chaperone binds to the hydrophobic tip of PPE41^11^,^12^. Heterologous co-expression of *espG*_*5*_ improved the solubility of the ESX-5 substrate pair PE31/PPE18, indicating that this protein acts as a classical chaperone by preventing protein aggregation^11^. Similarly, EspG_1_, the chaperone encoded by the *esx-1* locus, has been shown to bind only to its cognate substrate pair PE35/PPE68_1 and this component is essential for the solubility of the ESX-1 PE/PPE pair^14^. As EspG chaperones specifically recognize their cognate T7S substrates, they might be involved in determining system specificity.

In this study, we have elucidated the role of the EspG-binding domain of PPE proteins in system specificity in *M. marinum*, by replacing the EspG_1_ binding domain of the ESX-1 substrates PE35/PP68_1 with the EspG_5_ binding domain of the ESX-5 substrate PPE18.

## Results

### The EspG_1_ binding domain of PPE68_1 is important for secretion in *M. marinum*

**Figure 1:**
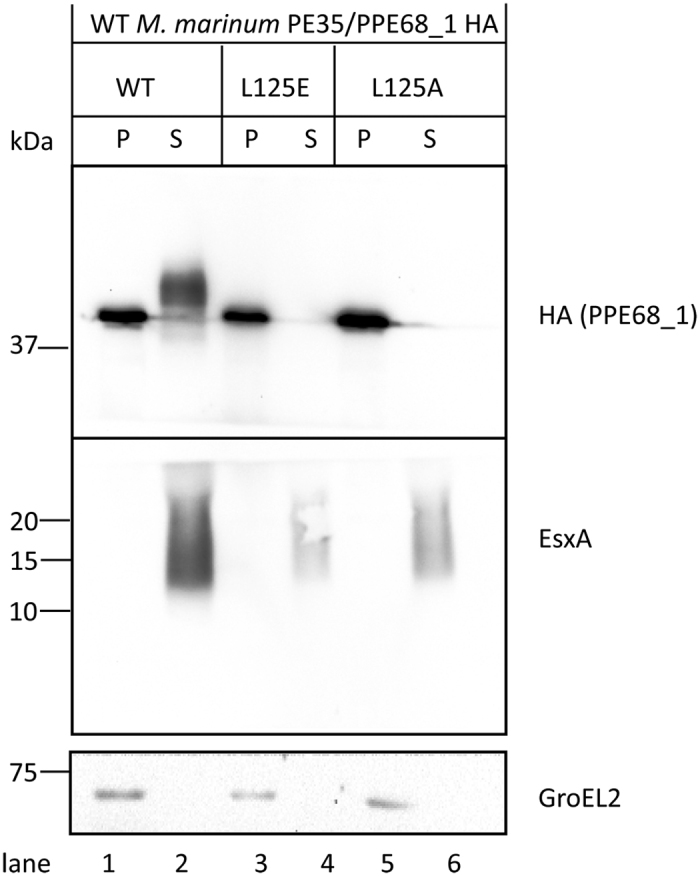
Effect of substitutions in the predicted EspG_1_ binding domain of PPE68_1 on the secretion of PE35/PPE68_1. Immunoblot analysis of bacterial pellets (P) and culture filtrates (S) of WT PPE68_1_HA and its variants L125E and L125A in the WT *M. marinum*, detected with an HA antibody. Intracellular GroEL2 and supernatant protein EsxA were detected as controls. Equivalent OD units were loaded; 0.2 OD for pellet and 0.5 OD for supernatant fractions.

In earlier studies, it was shown that the ESX-5 chaperone EspG_5_ binds to a conserved hydrophobic patch in the extended domain of PPE proteins^11^,^12^. Point mutations that changed this hydrophobic patch of the *M. marinum* ESX-5 substrates PPE18, which is secreted as a heterodimer together with PE31, and LipY abolished EspG_5_ binding and affected their secretion via the ESX-5 system^11^. This EspG binding region is relatively well conserved among PPE proteins of different ESX systems^11^, which allows the prediction of the EspG-binding motif of these proteins (Fig. S1). To investigate the role of the EspG-binding domains in system specificity, we first tested whether similar substitutions in the *M. marinum* ESX-1 substrate pair PE35/PPE68_1 had a similar effect on secretion. We created two point mutations in PPE68_1, *i.e.* L125E and L125A, and also modified the gene by adding a sequence coding for a C-terminal HA tag. Immunoblot analysis of bacterial pellets and culture filtrates using an anti-HA antibody showed that HA-tagged PPE68_1 (WT) was secreted as expected. The secreted protein had a slightly higher molecular weight than expected and appeared as a diffuse band (Fig. 1, lane 2). This behavior is seen for more ESX substrates and might be indicative of post-translational modifications. Variants with L125E and L125A substitutions were, in contrast to the WT control protein, produced but not secreted in the *M. marinum* WT strain (Fig. 1, lane 3, 4 and lane 5,6, respectively). This observation shows that, also for the ESX-1 substrate PE35/PPE68_1, this residue is crucial for secretion, presumably because of its importance for binding of the chaperone EspG_1_.

### PPE68_1 containing the EspG_5_-binding domain of PPE18 is secreted independent of ESX-1

**Figure 2:**
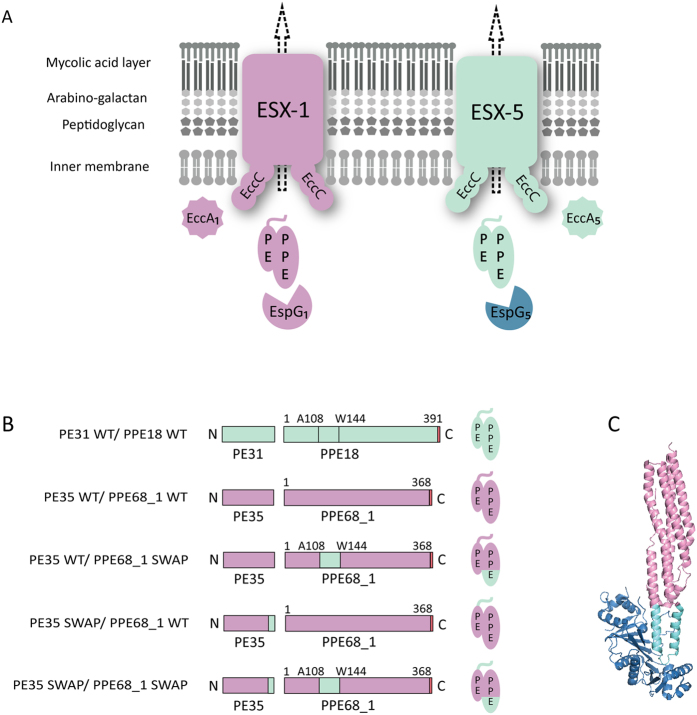
Model for substrate targeting and translocation in type VII secretion and a schematic representation of the different constructs used in this study. (**A**) The current model for substrate targeting in type VII secretion. The mycobacterial cell envelope is composed of an inner membrane and an outer mycolic acid-containing membrane. EspG chaperones specifically recognize their cognate PE/PPE substrates. The general YxxxD/E secretion signal at the C-terminus of PE proteins is exposed for interaction with the secretion machineries. EccC is one of T7S membrane components containing three ATP binding domains and is probably involved in substrate recognition by the systems. (**B**) Schematic representation of the WT *M. marinum* ESX-5 substrates PE31/PPE18 WT (in cyan), the WT *M. marinum* ESX-1 substrates PE35/PPE68_1 (in pink) and derivatives used in this research. The colors indicate the origin of the different domains and the HA-tag is shown in red. (**C**) A representation of the structure of EspG_5_ (in blue) bound to the ESX-5 substrate PE25/PPE41^11^. The corresponding EspG binding region that is replaced in this study is indicated in cyan, while the remained parts of the PE/PPE substrates are highlighted in pink.

The observation that the ESX-1 and ESX-5 system possess their own EspG chaperone that specifically binds to cognate PE/PPE substrates suggests that these chaperones might be involved in determining system specificity. We therefore tested whether it was possible to reroute an ESX-1 substrate to the ESX-5 system and *vice versa* by manipulating the cognate EspG-binding domains. We designed chimeric genes where the regions coding for 34 amino acids of PPE18 and PPE68_1, covering the entire predicted EspG_5_- and EspG_1_-binding domain (Fig. S1), respectively, were exchanged between the two *ppe* genes. Initial secretion analysis showed that PPE18 containing the EspG_1_-binding domain of PPE68_1 was not secreted in the WT *M. marinum* strain, although it was stably expressed (Fig. S2B). We therefore focused on the rerouting of PPE68_1. This ESX-1 substrate modified with the EspG_5_-binding domain of PPE18, was named PE35 WT/PPE68_1 SWAP (Fig. 2B and C). To additionally evaluate the role of the general secretion signal for determining system specificity of PPE68_1, the region encoding the C-terminal 15 amino acids, containing the YxxxD secretion motif, of the ESX-1 substrate PE35 was replaced by the similar region of ESX-5 substrate PE31 (Fig. S2 and Fig. 2B). This construct was designated PE35 SWAP/PPE68_1 WT. The hybrid protein pair, in which both the C-terminal secretion signal of PE31 and the predicted EspG_5_-binding domain of PPE18 were introduced, was named PE35 SWAP/PPE68_1 SWAP. These constructs were electroporated into the WT and various ESX-1 and ESX-5 mutant strains of *M. marinum* and the secretion profiles were assessed. Detection of cytosolic chaperone GroEL2 was included as a loading and lysis control.

**Figure 3:**
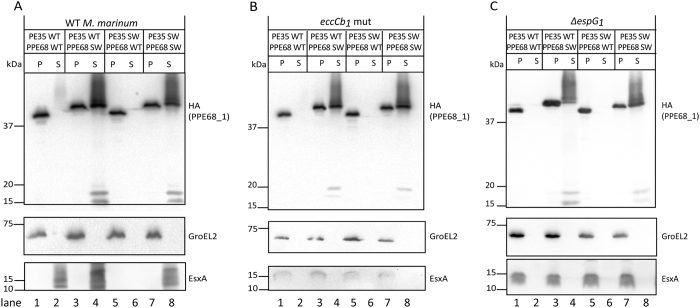
PPE68_1 containing the EspG_5_-binding domain is secreted in an ESX-1 independent manner. Immunoblot analysis visualizing the secretion of different PE35/PPE68_1 variants containing an HA-tag at the C-terminus of PPE68_1, using cell pellets (P) and culture supernatant (S) of WT *M. marinum* (**A**), *eccCb*_*1*_ mutant (**B**) and *∆*e*spG*_*1*_ (**C**) strains. GroEL2 and EsxA were used as loading controls. Equivalent OD units were loaded; 0.2 OD for pellet and 0.5 OD for supernatant fractions.

In the WT *M. marinum* strain, PPE68 WT was produced and secreted as a full length protein with an apparent molecular weight of ~40 kDa (Fig. 3A, lanes 1 and 2, respectively). Interestingly, PPE68_1 SWAP containing the EspG_5_-binding domain of PPE18, which runs slightly higher as compared to the original protein, was also secreted and the secretion efficiency was even substantially higher in comparison to that of PPE68_1 WT (Fig. 3A, lane 3 and lane 4, respectively). Moreover, PPE68_1 SWAP was detected in two secreted forms of about 17 kDa, which likely represents processed forms. An unexpected result was observed when we examined the single swap of the C-terminal secretion signal in PE35. This alteration did abolish the secretion of its partner protein PPE68_1 WT (Fig. 3A, lane 6). This phenotype was unexpected as a previous study showed that the C-terminal 15 amino acids of the ESX-1 and ESX-5 substrates are interchangeable without affecting the secretion of their PPE partners^13^. Possibly, the C-terminal secretion signals are not as interchangeable as previously reported. The same C-terminal replacement did not have any effect on secretion of the PPE partner, when the PE35 hybrid was expressed in combination with PPE68_1 SWAP (Fig. 3A, lane 8). This combination showed a similar phenotype as PE35 WT/PPE68_1 SWAP, suggesting that exchanging the secretion motif did not affect the secretion capability of the PPE68_1 containing the EspG_5_ binding domain. Together, these results show that PPE68_1 containing the EspG_5_-binding domain of PPE18 is efficiently secreted in the *M. marinum* WT strain, both with the presence of the ESX-1 or ESX-5 general secretion motif.

Interestingly, expression of PE35/PPE68_1 containing the single swap of the C-terminal secretion signal in PE35 abolished the secretion of another ESX-1 substrate EsxA (ESAT-6), as it was not detected in the culture supernatant nor in the pellet fraction. To address whether the lack of the EsxA signal was due to abolished e*sxA* transcription, total mRNA was extracted from *M. marinum* strain WT, an *eccCb*_*1*_ mutant (M^VU^, see below) and WT strains containing the four different constructs of PE35/PPE68_1. qRT-PCR was performed using three sets of primers which allowed for the specific detection of *esxA* cDNA. In the presence of PE35 SWAP, the expression level of *esxA* was still detected at a comparable level to that in the strains containing the other hybrid constructs (Fig. S3), showing that the loss of the EsxA signal was not due to altered transcription of the corresponding gene. We postulate that the PE35 SWAP construct frustrates ESX-1 functioning altogether, resulting in the degradation of EsxA.

Next, we determined which system was responsible for the secretion of PPE68_1 SWAP. Secretion was first analyzed in a *M. marinum* M^VU^ mutant strain, known to be deficient in ESX-1 secretion by a frameshift mutation in the *eccCb*_*1*_ gene^7^. EccCb_1_ is part of the EccC ATPase that is crucial for substrate recognition and transport^15^. We have previously shown that the secretion of the ESX-1 substrates EsxA and PPE68_1 was abolished in this mutant^13^, which is confirmed by the results shown in Fig. 3B, lane 2. In contrast to PPE68_1 WT, the PPE68_1 SWAP chimeric protein was produced and still efficiently secreted in this mutant (Fig. 3B, lanes 3 and 4, respectively). Also in this strain, the replacement of the C-terminal secretion signal of PE35 with the homologous sequence of the ESX-5 substrate PE31 did not affect the secretion of its partner, PPE68_1 SWAP (Fig. 3B, lanes 8). These data show that disruption of EccCb_1_ did not have an effect on the secretion of PPE68_1 SWAP. We subsequently investigated the involvement of the EspG_1_ chaperone in the secretion of PPE68_1 SWAP. For this, a targeted knockout strain of *espG*_*1*_ was made in a *M. marinum* WT (M^USA^) strain and we first examined the overall secretion defects of this new mutant. In contrast to the previously observed effect of knocking out *espG*_*1*_ in *M. tuberculosis*^16^, the deletion of *espG*_*1*_ in *M. marinum* had a profound effect on the secretion of ESX-1 substrates. Both EsxA and PPE68_1 WT were found in the pellet, but not secreted into the culture filtrate (Fig. 3C, lanes 1 and land 2, respectively), while the secretion of EspE, an ESX-1 cell surface localized substrate accumulating in the Genapol-X080 cell-surface extractable fraction^17^, was also blocked (Fig. S4, lane 6). The secretion of all these ESX-1 substrates was restored in the complemented *espG*_*1*_ knockout strain (Fig. S4, lane 7 and 8). When PPE68_1 SWAP was introduced into this strain, secretion was still efficient, as a full length form (~40 kDa) and two processed forms (~17 kDa) were observed regardless of the presence of an ESX-1 or ESX-5 C-terminal secretion signal (Fig. 3C, lane 4 and lane 8). These data show that PPE68_1 SWAP does not require the EspG_1_ chaperone for its secretion. In conclusion, the introduction of the EspG_5_-binding domain of PPE18 in the ESX-1 substrate PPE68_1 results in the ESX-1 independent secretion of this substrate.

### PPE68_1 containing the EspG_5_-binding domain is rerouted to the ESX-5 system

**Figure 4:**
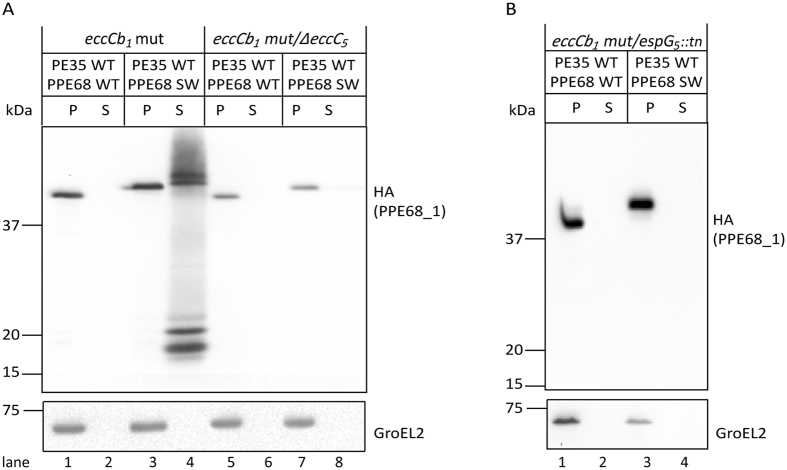
PPE68_1 carrying the EspG_5_-binding domain is rerouted to the ESX-5 system. Immunoblot analysis visualizing the secretion of the PE35/PPE68_1 variants using cell pellets (P) and culture supernatants (S) of the *M. marinum eccCb*_*1*_ mut/*∆eccC*_*5*_ (**A**) and the *eccCb*_*1*_ mut/*espG*_*5*_*::tn* (**B**) double mutant strains. The WT and mutated variants of PE35/PPE68_1_HA were detected with an HA antibody. GroEL2 was included as cytosolic control. Equivalent OD units were loaded; 0.2 OD for pellet and 0.5 OD for supernatant fractions.

The above observations suggest that PPE68_1 SWAP now relies on the ESX-5 system for its secretion. To test this, the secretion of this hybrid protein was investigated in several ESX-5 mutant strains. First, the involvement of EccC_5_ in the secretion of the SWAP construct was assessed. For this, we used the previously characterized double mutant strain of *M. marinum* that contains the frameshift mutation of *eccCb*_*1*_, the M^VU^ strain, and a targeted deletion of *eccC*_*5*_^18^. This double mutant has previously been shown to be defective in the secretion of the ESX-5 substrates EsxN and the PE_PGRS proteins^18^. Similarly as observed in the *eccCb*_*1*_ mutant strain, although PPE68_1 WT was detected in the pellet, the protein was not secreted in the *eccCb*_*1*_/*eccC*_*5*_ double mutant strain (Fig. 4A, lanes 5 and 6). Interestingly, PPE68_1 SWAP, while being efficiently secreted in the *eccCb*_*1*_ mutant (Fig. 4A, lane 4), was found in the pellet fraction and no longer in the culture filtrate (Fig. 4A, lanes 7 and 8). These data suggest that the SWAP construct is secreted via the ESX-5 membrane channel.

To investigate the role of the EspG_5_ chaperone in the secretion of PE35/PPE68_1 SWAP, we used a mutant in which *espG*_*5*_ was disrupted by a transposon insertion (*espG*_*5*_*::tn*) in the *eccCb*_*1*_ mutant (M^VU^) background. The secretion of PE_PGRS proteins has been shown earlier to be abolished in this double mutant strain^6^, which was restored by introduction of a complementation construct (Fig. S5). As expected, PPE68_1 WT was not secreted in this strain (Fig. 4B, lane 2). Importantly, also the secretion of PPE68_1 SWAP was blocked in this strain, as it was only detected in the pellet fraction (Fig. 4B, lanes 3 and 4). This result shows that EspG_5_ is essential for the secretion of the chimeric PPE68_1 SWAP. Together, we conclude that PPE68_1 containing the EspG_5_ binding domain is fully dependent on the ESX-5 system for secretion.

### PE35/PPE68_1 SWAP affects the secretion of endogenous ESX-5 substrates

**Figure 5:**
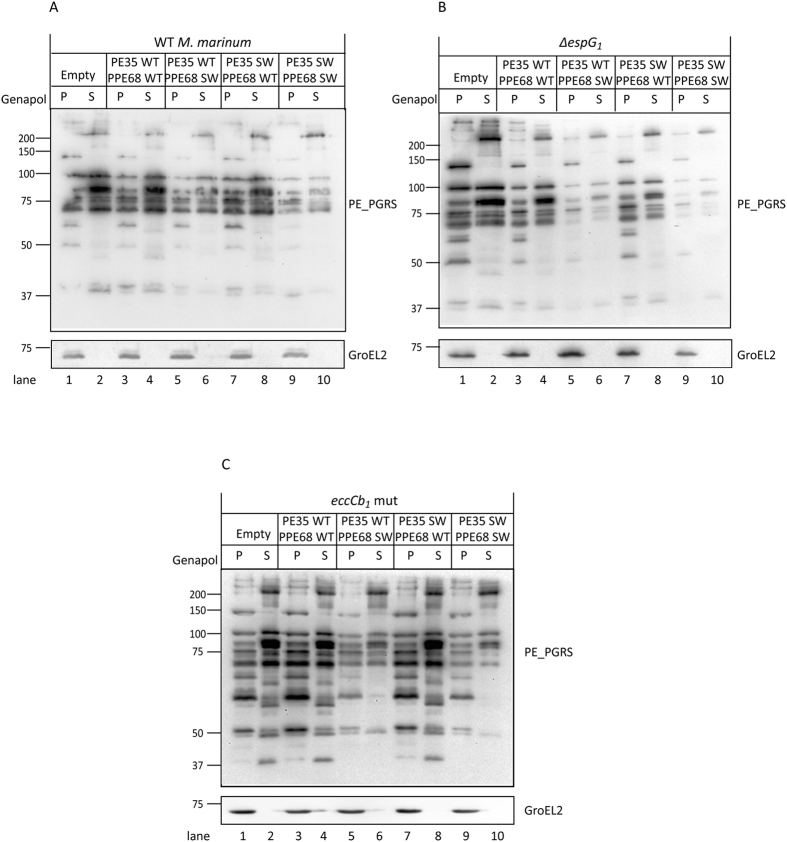
The rerouting of PE35/PPE68_1 SWAP to the ESX-5 system affects the secretion of endogenous ESX-5 substrates. Surface localization of the ESX-5 dependent PE_PGRS substrates in the WT *M. marinum* (**A**), *∆espG*_*1*_ strain (**B**) and the *eccCb*_*1*_ mutant strain (**C**) carrying various PE35/PPE68_1 variants was analyzed by immunoblotting. Bacterial cell pellets (P) were treated with Genapol X-080 to enrich for capsular proteins (S). Equivalent OD units were loaded; 0.2 OD for pellet and 0.5 OD for Genapol X-080 treated surface fractions. GroEL2 was included as loading control.

Finally, we examined whether the rerouting of PPE68_1 SWAP to the ESX-5 system had any influence on the secretion of endogenous ESX-5 substrates, the PE_PGRS proteins. For this, the secretion of ESX-5 dependent PE_PGRS proteins was analyzed in *M. marinum* WT, *∆espG*_*1*_ and *eccCb*_*1*_ mutant strains expressing the four different constructs of PE35/PPE68_1. Interestingly, in the WT strain, where PPE68_1 SWAP is rerouted to the ESX-5 system, a significant decrease in PE_PGRS signal was observed in both the cell pellet and the cell surface-enriched fraction (Fig. 5A, lanes 5, 6 and lane 9, 10). Similar phenotypes were also observed in the *∆espG*_*1*_ and *eccCb*_*1*_ mutant strains (Fig. 5B,C, lanes 5, 6 and lane 9, 10). In contrast, the overexpression of the ESX-1 dependent substrates PPE68 WT had no effect on the intracellular level and secretion of PE_PGRS proteins (Fig. 5A,B and C, lane 3, 4 and lane 7, 8). These results suggest that there is a competitive correlation between the secretion of the rerouted substrates PE35/PPE68_1 SWAP and native substrates of ESX-5 system, the PE_PGRS proteins.

## Discussion

Mycobacteria possess multiple ESX system, each of which secretes a specific subset of Esx and PE/PPE substrates. This dictates that T7S substrates should possess system-specific recognition signals^14^. Previously, it was observed that exchanging the general YxxxD/E secretion motif between an ESX-1 and ESX-5 PE/PPE substrate pair did not affect system specificity^13^, showing that these substrates should contain an additional recognition signal that determines through which ESX system they are transported. In this study, we demonstrate that, by replacing the putative EspG_1_-binding domain of the ESX-1 substrate PPE68_1 with the analogous domain of the ESX-5 substrate PPE18, this substrate is redirected to the ESX-5 system in *M. marinum*. This shows that this domain is a crucial determinant for defining system specificity.

Interestingly, the redirected PE35/PPE68_1 chimeric protein pair is even more efficiently secreted via the ESX-5 system as compared to secretion of the original heterodimer via the ESX-1 system. Possibly, ESX-5 is more active in *M. marinum* as compared to ESX-1, which is in line with previously obtained proteomic data that ESX-5 components are more abundantly present in the cell envelope of this species^6^. The efficient secretion of the hybrid substrate pair resulted in less intracellular production and secretion of certain endogenous ESX-5-dependent substrates, suggesting that the exogenous protein pair efficiently competes with endogenous substrates for recognition and export. Our data support the model in which EspG not only functions as a chaperone that keeps PE/PPE substrates in a translocation competent state, but that this protein also targets substrates to the cognate translocation machinery. This is not unique, as chaperones of other bacterial protein secretion systems have been shown to play multiple roles. For example, SycE and SycO of the type III secretion system are required for both substrate stabilization and targeting of the substrate to the transport recognition site^19^,^20^. A role in substrate stabilization and targeting towards the secretion channel was also shown for FimC and PapD chaperones in the chaperon-usher pathway^21^.

Previously, it has been proposed that there may be cross-talk between different ESX systems and that T7S substrates are not strictly dependent on the cognate EspG chaperone for secretion^12^. Here, we show that secretion of PE/PPE protein pairs by ESX-1 and ESX-5 is strongly affected in the absence of the cognate EspG chaperone, showing that for these substrates there is no redundancy in EspG functioning. We additionally observed a defect in EsxA secretion in the *espG*_*1*_ mutant in *M. marinum*, which is similar as the previously observed secretion defects of the ESX-5 dependent EsxN in *M. marinum espG*_*5*_ mutant strains^7^. These results show that EspG chaperones are not only involved in secretion of PE/PPE proteins, but also in the secretion of Esx substrates in *M. marinum*. Because Esx proteins do not have a typical EspG binding domain, this effect is probably indirect. Indeed, ESX-1 substrates are well known for being interdependent for secretion^22^. Previous research indicated that in *M. tuberculosis*, EsxA was still secreted after deletion of *espG*_*1*_^16^. The role of EspG in the secretion of Esx proteins might therefore vary between different mycobacterial species or that there is redundancy in EspG functioning in *M. tuberculosis*. In the same study, expression of PPE68, which is not secreted but remains cell envelope associated in *M. tuberculosis*, appeared to be reduced in the absence of EspG_1_, which might be indicative of the role of this chaperone in the proper cell envelope localization of this PPE protein in this species.

An unexpected observation was that when the ESX-1 substrate PE35 carried the secretion motif of the ESX-5 substrate PE31, PPE68_1 WT was not secreted anymore. Strikingly, the presence of this hybrid protein pair also completely abolished the presence of endogenous EsxA in the culture filtrate and bacterial pellet fractions, suggesting that the general functioning of the ESX-1 system is blocked. Because the transcript levels of *esxA* in this strain was comparable to that in other conditions, we reasoned that the absence of EsxA is likely triggered by incompatibility of secretion signals on EsxA/B and PE35 SWAP/PPE68_1. Recently, a study by Rosenberg *et al*. showed that EsxB binding was required for multimerization and activation of EccC^15^. A model was subsequently proposed in which simultaneous binding of multiple substrates to the ATPase ensures coordinated interdependent secretion of these substrates. Therefore, dual binding of EsxB and PE35 SWAP to EccCab_1_ could block proper activation and functioning of the ESX-1 system. Importantly, the secretion block of PPE68_1, caused by the introduction of the PE31 secretion signal, was overcome by the introduction of EspG_5_-binding domain in PPE68_1, resulting in secretion via the ESX-5 system. The secretion of this double-swap construct via ESX-5 was as efficient as the construct only containing the EspG binding domain swap. The origin of the general secretion signal is possibly less important for proper recognition and secretion via ESX-5 compared to ESX-1. Related to this is the observation that introduction of the predicted EspG_1_ binding domain of PPE68_1 into the ESX-5 substrate PPE18 abrogated secretion of this hybrid protein in WT *M. marinum*. Although this hybrid protein could be misfolded, hindering its secretion, another cause could be that the ESX-1 system is less flexible in accommodating various substrates. We postulate that the flexibility in substrate recognition by ESX-5 is required to recognize and transport the large number of highly variable ESX-5 dependent PE/PPE substrates, while the ESX-1 system only secretes a limited number of these proteins.

The co-dependent secretion of many ESX-1 substrates^22^ makes it highly complicated to study their individual role during infection. The successful rerouting of an ESX-1 substrate pair to the ESX-5 system in this study provides a promising method to investigate these different roles. In this study, we have applied this approach to study the functional role of PE35/PPE68_1 in the ability of mycobacteria to lyse erythrocytes, for which a functional ESX-1 system is required^23^,^24^. While WT *M. marinum* showed a clear contact-dependent lysis of erythrocytes in a hemolysis assay (Fig. S6), the *eccCb*_*1*_ mutant showed hardly any hemolytic activity. The ESX-5 dependent secretion of PPE68_1 SWAP could not induce any hemolytic activity in this mutant strain. We could therefore conclude that this protein has no membrane lysing abilities. Finally, the current live BCG vaccine strain is attenuated mainly due to deletion of a large part of the ESX-1 secretion system and is thus unable to secrete any ESX-1 substrates^25^. The loss of the ESX-1 system and the subsequent inability to survive in macrophages probably resulted in over-attenuation of this strain to be an effective vaccine. The rerouting approach in this study could be used to improve the vaccine strain, by redirecting specific ESX-1 substrates that induce a strong immune response and/or enhance intracellular survival.

## Materials and Methods

### Bacterial strains and cultural conditions

*M. marinum* wild-type strain M^USA^ and mutant derivatives were cultured at 30 °C in Middlebrook 7H9 liquid medium or Middlebrook 7H10 agar supplemented with 10% Middlebrook ADC and 0.05% Tween 80. *E. coli* TOP10F’ strain was grown at 37 °C in LB medium and was used for cloning experiments and generating plasmid DNA. When needed, antibiotics were added to the cultures at the following concentrations: kanamycin, 25 μg/ml, streptomycin, 30 μg/ml, hygromycin, 50 μg/ml for mycobacteria and 100 μg/ml for *E. coli*.

### Plasmid construction

All DNA manipulations were carried out according to standard procedures. In short, PCR amplifications of target genes were performed using Phusion^®^ High-Fidelity DNA Polymerase (Finnzymes) with chromosomal DNA from *M. marinum* M^USA^ or plasmid DNA as templates. The pSMT3 *E. coli*-mycobacterial shuttle vector was used as a recipient plasmid for PE35-PPE68_1 and PE31-PPE18. Anchored primers designed to contain a HA-tag at the C-terminus of the genes were used to amplify the targeted genes from chromosomal DNA of *M. marinum* M^USA^ as a template using Phusion^®^ High-Fidelity DNA Polymerase (Finnzymes). Amplicons were cloned as NheI-BamHI fragments (*pe35/ppeE68_1*) or SpeI_BamHI (*pe31/ppe18*) into NheI-BamHI digested pSMT3, resulting in pSMT3::*pe35/ppe68_1_HA* and pSMT3::*pe31/ppe18_HA.* The swap constructs and point mutations were synthesized by nested PCR with appropriate primers. To clone the desired genes into a derivative of pMV361^26^, the EcoRI-HindIII digested fragments of *pe35/ppe68_1* WT and *pe35/ppe68_1* SWAP were ligated to the EcoRI-HindIII digested pMV, resulting in pMV::*pe35/ppe68_1* WT and pMV::*pe35/ppe68_1* SWAP. To create a version of the obtained plasmids containing a streptomycin resistance cassette, the kanamycin resistance cassette was exchanged by a streptomycin resistance cassette by digesting HindIII-PciI. All constructs were confirmed by DNA sequencing and introduced into *M. marinum* strains by electroporation. All plasmids are described in Table S2 and primers are listed in the Table S3.

### Generating an *espG*_*1*_ knock-out in *M. marinum*

To create the *espG*_*1*_ knock-out in *M. marinum*, the previously described approach was followed^27^. For deletion of *espG*_*1*_, PCR was performed to synthesize fragments bearing the 1398 and 1083 bps of flanking regions of endogenous *espG*_*1*_ of *M. marinum*, resulting in a deletion of 92% of the gene (primer set EspG1 KO LF and EspG1 KO LR for the 5′ region and primer set EspG1 KO RF and EspG1 KO RR for the 3′ flanking region). Amplicons corresponding to upstream and downstream flanking regions were digested with Van91I and cloned into the Van91I digested p0004s plasmid that contains a hygromycin resistance cassette and the *sacB* gene to be able to select for sucrose sensitivity. This allelic exchange substrate was introduced into the PacI site of phasmid phAE159 and electroporated into *M. smegmatis* mc^2^155 to obtain high titers of phage pHAE159 28. Subsequently, the *M. marinum* wild-type strain was incubated with high titers of corresponding phage to create *espG*_*1*_ knockouts. Colonies that had deleted the endogenous *espG*_*1*_ were selected on hygromycin plates and verified for sucrose sensitivity. The deletion was confirmed by PCR analysis and sequencing. To remove the resistance gene, a temperature sensitive phage encoding the γδ-resolvase (TnpR) (a kind gift from Apoorva Bhatt, University of Birmingham, UK) was used, generating an unmarked deletion mutation. To complement the knockout strain of *espG*_*1*_, the PacI-HindIII digested fragment of *MMAR_5441*/*MMAR_5442* /*MMAR_5443* was ligated to the compatibly digested pMV, resulting in pMV::*MMAR_5441*/*MMAR_5442* /*MMAR_5443 (Km*^*R*^).

### Protein secretion and immunoblotting

The analysis of *M. marinum* protein secretion was carried out as previously described^13^. For immunblot analysis, the membranes were stained with mouse monoclonal antibodies against the influenza hemagglutinin epitope (HA.11, Covance), anti-GroEL2 (CS44, John Belisle, NIH, Bethesda, MD, USA), anti-ESAT6 (Hyb 76-8; Statens Serum Institut, Copenhagen, Denmark) and mouse anti-PE_PGRS (7C4.1F7 7). The secondary horseradish peroxidase conjugated antibodies included goat anti-mouse IgGs (A106PS, American Qualex) or goat anti-rabbit IgDs (611-1302, Rockland), which were detected with chemiluminescence (Pierce).

### Hemolysis

Hemolysis experiments were performed in the *M. marinum* WT, *eccCb*_*1*_ mutant and *eccCb*_*1*_ mutant overexpressed PE35/PPE68_1 SWAP as previously described^18^,^29^.

### RNA isolation and quantitative RT-PCR

RNA was purified from bacterial cultures grown to an OD600 of 1. Bacteria were lysed by bead-beating in the presence of TRIzol Reagent (Invitrogen), and RNA was isolated using Mackery-Nagel NucleoSpin RNA isolation kit. RNA samples were eluted in water with Ribolock (Thermoscientific). cDNA was synthesized from 400 ng total RNA with the SuperScript VILO cDNA synthesis kit (Invitrogen), according to the protocol supplied by the manufacturer. cDNA was diluted 1:10 in water prior to use in subsequent PCR reactions. Reactions were set up using EXPRESS SYBR GreenER qPCR SuperMix Universal (Invitrogen), including the addition of ROX dye reference. Quantitative RT-PCR was performed with the ABI 7500 Real-Time PCR System (Applied Biosystems). At the end of amplification, PCR product specificity was verified by dissociating curve analysis and agarose gel electrophoresis. The threshold cycle (Ct) values are normalized with Ct of *sigA*.

## Additional Information

**How to cite this article**: Phan, T. H. *et al*. Identification of a substrate domain that determines system specificity in mycobacterial type VII secretion systems. *Sci. Rep.* **7**, 42704; doi: 10.1038/srep42704 (2017).

**Publisher's note:** Springer Nature remains neutral with regard to jurisdictional claims in published maps and institutional affiliations.

## Supplementary Material

Supporting Information
