# Supplementary material for: Identification of a substrate domain that determines system specificity in mycobacterial type VII secretion systems
Source: Sci Rep. 2017 Feb 16;7:42704. doi: 10.1038/srep42704 (PMC5311947; doi:10.1038/srep42704)
Supplement: Supporting Information [file srep42704-s1.pdf]

Identification of a substrate domain that determines system specificity in mycobacterial type VII secretion systems

Trang H. Phan[1], Roy Ummels[2], Wilbert Bitter[1,2], Edith N.G. Houben[1*]

[1]Section Molecular Microbiology, Amsterdam Institute of Molecules, Medicines & Systems, Vrije Universiteit Amsterdam, Amsterdam, The Netherlands

[2]Department of Medical Microbiology and Infection Control, VU University Medical Center, Amsterdam, The Netherlands

* Corresponding author. Email: e.n.g.houben@vu.nl

Supporting information

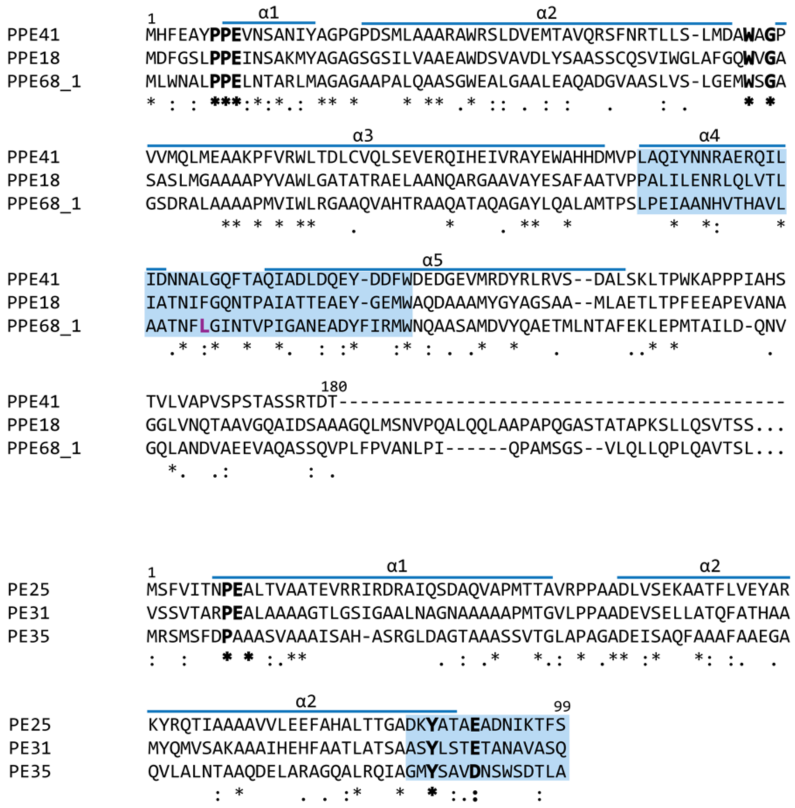

Figure S1: Sequence alignments of PPE and PE proteins used in this study. The conserved PPE and WxG motifs in PPE proteins and PE and secretion signal YxxxD/E motifs in PE proteins are depicted in bold. In the PPE sequence alignment, blue regions are the predicted EspG binding domains that were swapped in this study. The residue L125 of PPE68_1 is bolded and indicated in purple. In the PE sequence alignment, the blue regions indicate the C-terminal 15 amino acids, containing the YxxxD/E motifs that were swapped between ESX-1 and ESX-5 PE substrates in this study.

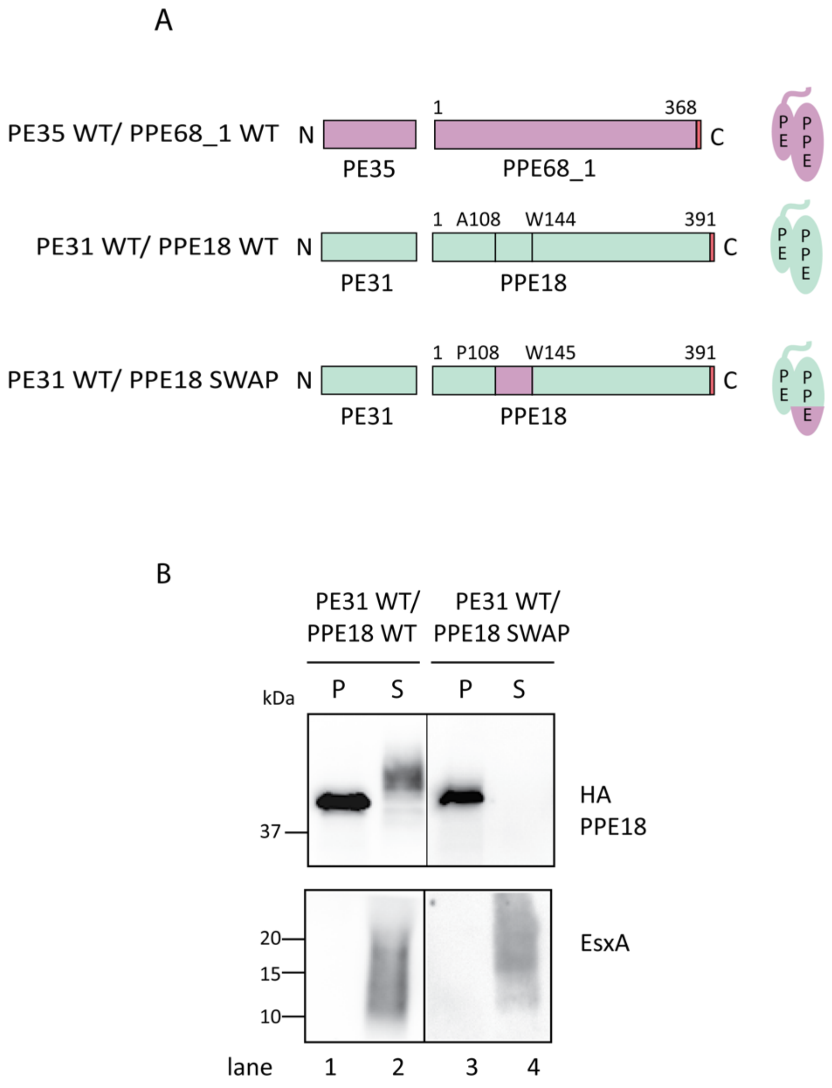

Figure S2: PPE18 containing the predicted $EspG_1$ binding domain of PPE68_1 is not secreted in the *M. marinum* WT strain. A, Schematic representation of the WT *M. marinum* ESX-5 substrates PE31/PPE18 WT (in cyan), the WT *M. marinum* ESX-1 substrates PE35/PPE68_1 (in pink). The predicted $EspG_1$ binding region of PPE68_1 (P108-W145) was introduced into the corresponding region of PPE18 (A108-W144), resulting in PPE18 SWAP. **B**, Immunoblot analysis of PPE18 WT and PPE18_SWAP secretion by overexpression of *ppe18* and *ppe18 swap* on the episomal plasmid pSMT3 under the control of the *hsp60* promoter. P=bacterial pellet, S=culture filtrate.

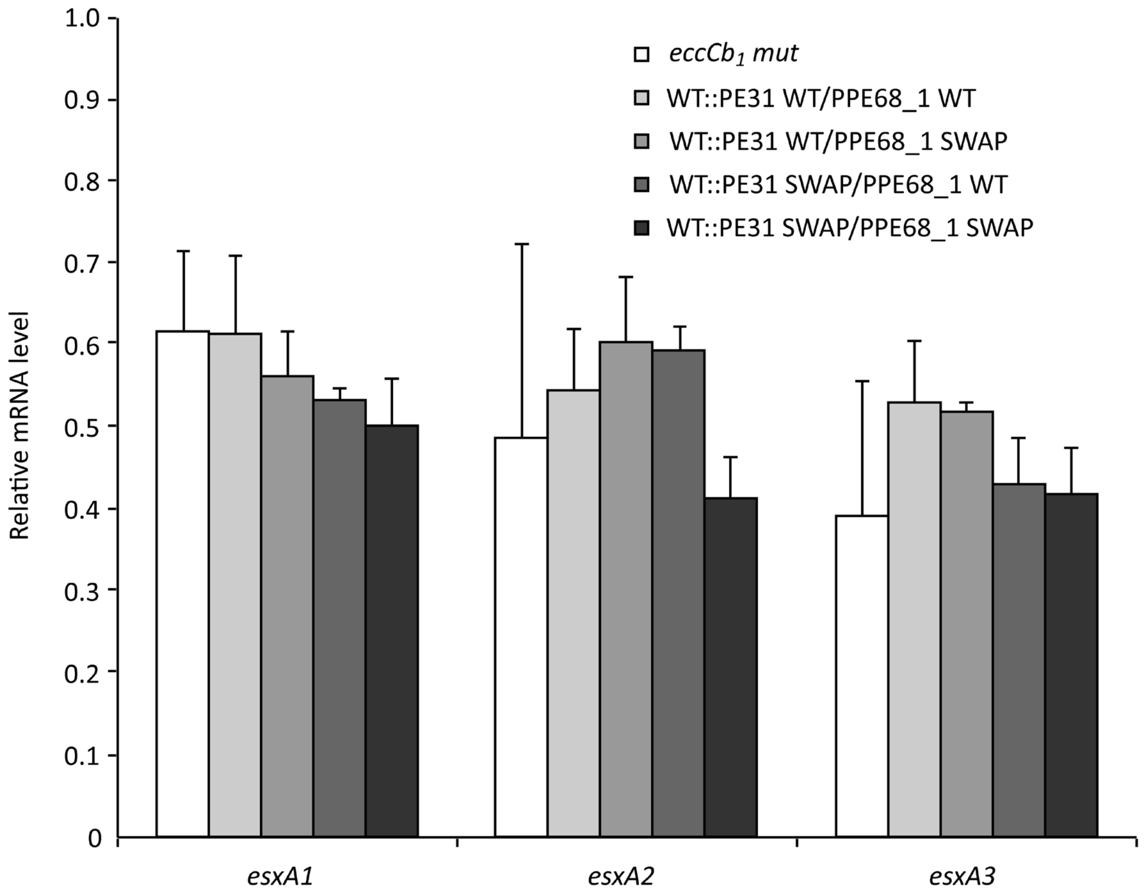

Figure S3: Effect of different PE35/PPE68_1 hybrid constructs on the transcript level of *esxA*. Total RNA was isolated from WT *M. marinum* M^{USA}, *eccCb*$_1$ mutant strain, and M^{USA} strains exogenously expressing PE35 WT/PPE68_1 WT, PE35 WT/PPE68_1 SWAP, PE35 SWAP/PPE68_1 WT, PE35 SWAP/PPE68_1 SWAP. Three different sets of primers, including *esxA1*, *esxA2* and *esxA3*, were used to amplify *esxA* cDNA. Ct values were normalized for Ct values of the household gene *sigA* and compared to Ct values of *esxA* obtained from WT M^{USA}.

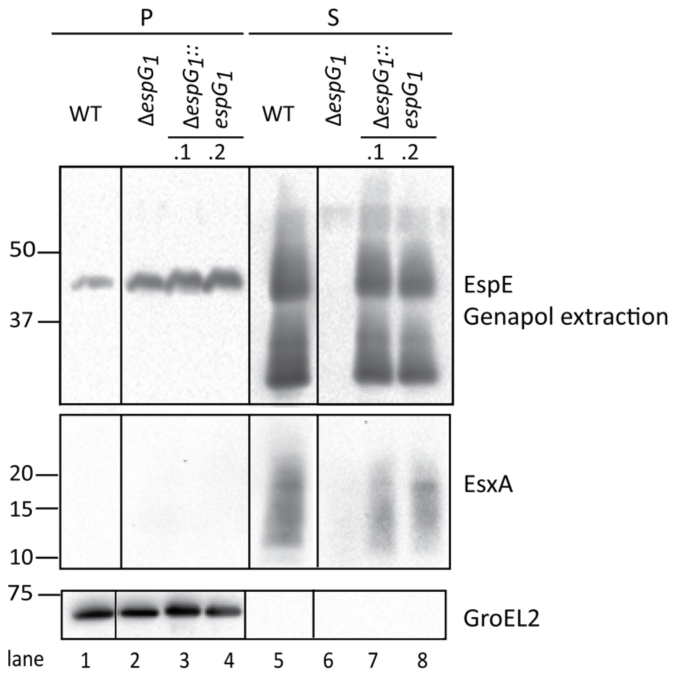

Figure S4: Effect of *espG₁* knockout and its complementation on the secretion of ESX-1 substrates. Secretion analysis in the *ΔespG₁* strain and corresponding complemented variant harboring the complete region of *espH-espG₁-eccA₁* on an integrative plasmid pMV under the control of the *hsp60* promoter. P=bacterial cell lysates, S=culture filtrate. Surface localization of the secreted EspE was restored upon an introduction of *espG₁* as it was found in the Genapol X-080 cell extraction, whereas EsxA was re-secreted into the cultural supernatant. GroEL2 was included as the cytosolic and supernatant control. 0.2 OD unit of Genapol pellet and 0.5 OD unit of Genapol and culture supernatant were loaded. P=bacterial cell lysates, S=culture filtrate.

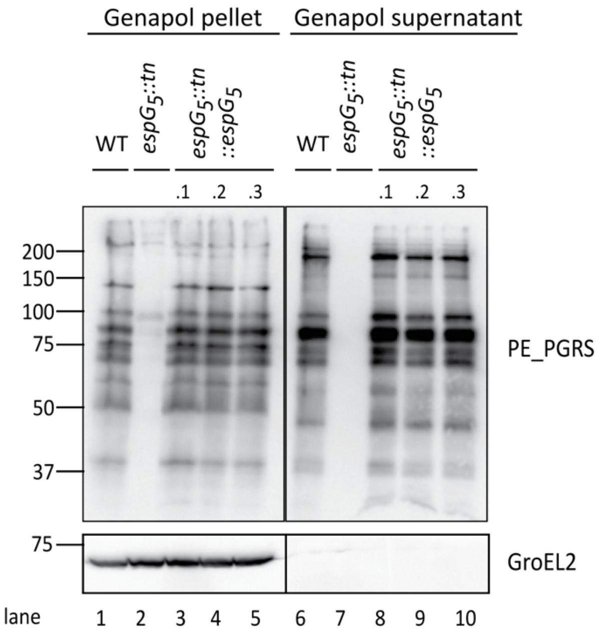

Figure S5: Restoration of PE_PGRS protein secretion by complementing the $espG_5$ transposon mutant strain. $eccCb_1$ mut/$espG_5$::*tn* was complemented with the integrative pMV plasmid containing the *esxM*-*esxN*-$espG_5$ gene cluster and a streptomycin resistance gene. Fractions enriched for cell-surface proteins of M^{USA}, $eccCb_1$ mut/$espG_5$::*tn* and complemented variant were isolated by the Genapol X-080 extraction and analyzed by western blot analysis. PE_PGRS signals were found in the pellet of $espG_5$::*tn* strain upon the complementation of $espG_5$ and its cell-surface localization was restored. GroEL2 was included as loading control. 0.2 OD unit of Genapol pellet and 0.5 OD unit of Genapol supernatant were loaded.

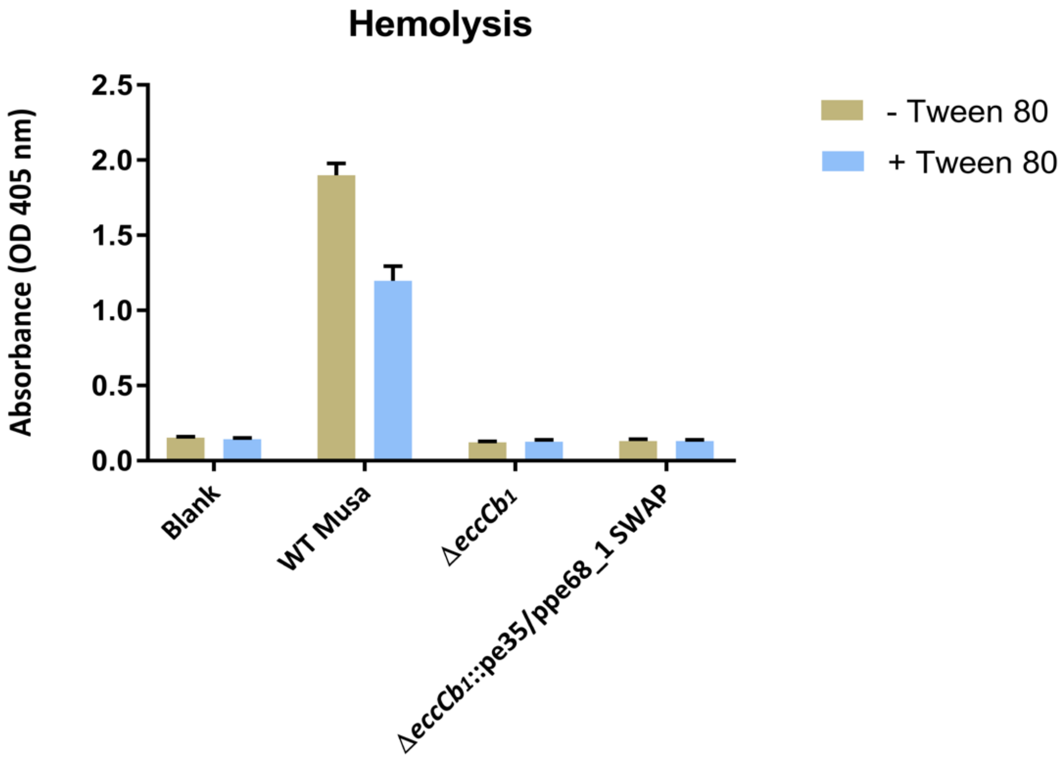

Figure S6: PPE68_1 SWAP has no hemolytic activity. *M. marinum* M[USA] wild-type, *eccCb*$_1$ mutant and *eccCb*$_1$ mutant secreting PPE68_1 SWAP were grown in the presence or absence of Tween 80. Subsequently, washed bacterial cells were used for the hemolysis assay.

Table S1: Strains used in this study

**Strains**	**Characteristics**	**Reference**
E. coli		
TOP10F’	F’[$lacI^q$, *Tn10*(TetR)] *mcrA* (*mrr-hsdRMS-mcrBC*) ϕ80*lacZ*ΔM15 *ΔlacX74 recA1 araD139* Δ *(ara leu)* 7697 *galU galK rpsL* (StrR) *endA1 nupG*; Tc^R	Invitrogen
M. marinum		
M^{USA}	Wild-type strain	[30]
M^{VU}	M^{USA} with single base insertion in e$ccCb_1$ (*MMAR_5446*)	[7]
M^{VU}/ Δ$eccC_5$	M^{VU} with unmarked eccC_5 (*MMAR_2665*) deletion. The strain additionally contains the plasmid pSMT3::*mspA* for viability	[27]
Δ$espG_1$	M^{USA} with the unmarked $espG_1$ (*MMAR_5441*) deletion	This study
LA1	M^{VU} with mariner transposon inserted in $espG_5$ (*MMAR_2676*). The strain additionally contains the plasmid pSMT3::*mspA* for viability	[27]

Table S2: Plasmids used in this study

Plasmids	Characteristics	Reference
pSMT3::PE31/PPE18_HA	pSMT3 carrying in SpeI-BamHI a 1.56-Kb PCR fragment containing PE31 (*MMAR_4241*) and PPE18 (*MMAR_4240*) genes with HA-tagged C terminus, Hyg^{R}	This study
pSMT3::PE35/PPE68_1_HA	pSMT3 carrying in NheI-BamHI a 1.49-Kb PCR fragment containing PE35 (*MMAR_0185*) and PPE68_1 (*MMAR_0186*) genes with HA-tagged C-terminus, Hyg^{R}	This study
pSMT3::PE31/PPE18 SWAP_HA	pSMT3 carrying in SpeI-BamHI a 1.6-Kb PCR fragment containing PE31 (*MMAR_4241*) gene and C-terminally tagged PPE18 (*MMAR_4240*) with a region of P107_W145 being replaced by PPE68_1 L106_W144, Hyg^{R}	This study
pSMT3::PE35/PPE68_1 SWAP_HA	pSMT3 carrying in NheI-BamHI a 1.49-Kb PCR fragment containing PE35 (*MMAR_0185*) gene and C-terminally tagged PPE68_1 (*MMAR_0186*) with a region of P107_W144 being replaced by PPE18 A108_W144, Hyg^{R}	This study
pSMT3::PE35 SWAP/PPE68_1_HA	pSMT3 carrying in NheI-BamHI a 1.49-Kb PCR fragment containing PE35 with a region of 15 amino acid PE31 C-terminal secretion signal and C-terminally HA-tagged PPE68_1, Hyg^{R}	This study
pSMT3::PE35 SWAP/PPE68_1 SWAP_HA	pSMT3 carrying in NheI-BamHI a 1.49-Kb PCR fragment containing PE35 swapped with a region of 15 amino acid PE31 C-terminal secretion signal and C-terminally tagged PPE68_1 with a region of P107_W144 being replaced by PPE18 A108_W144, Hyg^{R}	This study

pMV::PE35/PPE68_1_HA (Kan^{R})	pMV carrying EcoRI-HindIII a 1.49-Kb PCR fragment containing PE35 (*MMAR_0185*) and PPE68_1 (*MMAR_0186*) genes with HA-tagged C-terminus, Kan^{R}	This study
pMV::PE35/PPE68_1 SWAP_HA (Kan^{R})	pMV carrying EcoRI-HindIII a 1.49-Kb PCR fragment containing PE35 (*MMAR_0185*) and a C-terminally HA-tagged PPE68_1 (*MMAR_0186*) genes with a region of P107_W144 being replaced by PPE18 A108_W144, Kan^{R}	This study
pMV::PE35/PPE68_1_HA (Strep^{R})	pMV carrying PE35 (*MMAR_0185*) and PPE68_1 (*MMAR_0186*) genes with HA-tagged C-terminus, Strep^{R}	This study
pMV::PE35/PPE68_1 SWAP_HA (Strep^{R})	pMV carrying PE35 (*MMAR_0185*) and PPE68_1 (*MMAR_0186*) genes with HA-tagged C-terminus with a region of P107_W144 being replaced by PPE18 A108_W144, Strep^{R}	This study
pSMT3::PE35/PPE68_1 L125E	pSMT3::PE35/PPE68_1 in which leucine-125 has been mutated into a glutamine, Hyg^{R}	This study
pSMT3::PE35/PPE68_1 L125A	pSMT3::PE35/PPE68_1 in which leucine-125 has been mutated into an alanine, Hyg^{R}	This study
pMV::*espG$_1$/espH/eccA$_1$* (Kan^{R})	pMV carrying PacI-HindIII an *espG$_1$/espH/eccA$_1$* (*MMAR_5441*/*MMAR_5442* /*MMAR_5443*), Kan^{R}	This study
pMV::*esxM/esxN/espG$_5$* (Strep^{R})	pMV carrying XmnI-HindII an *M. marinum* *esxM/esxN/espG$_5$*, Strep^{R}	This study

Table S3: Primers used in this study

Plasmids	Primer name	Primer sequence	Restriction site
pSMT3::PE31/PPE18_HA	PE31-SPEI-F<br>PPE18-HA-BAMHI-R	CCCACTAGTGTGTCTTCTGTTACGGCTC<br>CCCGGATCCTCACGCGTAGTCCGGCACGTCGTACGGGTA TCCCGCGGCTGGAGGG	SpeI<br>BamHI
pSMT3::PE35/PPE68_1_HA	PE35-NHEI-F<br>PPE68_1-HA-BAMHI-R	CCCGCTACG ATGCGATCCATGTCTTTTGA<br>CCCGGATCCTCACGCGTAGTCCGGCACGTCGTACGGGTA CCAGTCGTCGTCGTCATCC	NheI<br>BamHI
pSMT3::PE35/PPE68_1 L125R_HA	PPE68_1 L125R-F<br>PPE68_1 L125R-R	GACCAATTTCCGAGGCATCAACA<br>TGTTGATGCCTCGGAAATTGGTC	
pSMT3::PE35/PPE68_1 L125A_HA	PPE68_1 125A-F<br>PPE68_1 125A-R	GACCAATTTCCGCCGCATCAACAC<br>GTGTTGATGCGGCGGAAATTGGTC	
pSMT3::PE35/PPE68_1 SWAP_HA	PPE18 A108_W144_Fw<br>PPE18 A108_W144_Rv<br>PPE68_L106_Rv<br>PPE68 N145_Fw	CCTTGGCGATGACGCCGTCGCTGGCGTTGATCCTCGAAAACC<br>CATCGCGCTGGCGGCCTGGTTCCACATCTCGCCGTACTCAG<br>GGATCAACGCCAGCGACGGCGTCATC<br>CGAGATGTGGAACCAGGCCGCCAGC	NheI<br>BamHI
pSMT3::PE35 SWAP/PPE68_1_HA	PE35_PE31(15C) ss_Fw<br>PE35_PE31(15C) ss_Rv	GAAACCGCCAACGCCGTGGCATCTCAA TAGTCGGCCTGCCAAC<br>TTGAGATGCCACGGCGTTGGCGGTTTCGGTGCTCAGGTACGAGGCCGCGATCTGCCGC	NheI<br>BamHI
pMV::PE35/PPE68_1_HA<br>pMV::PE35_PPE68_1 SWAP_HA	PE35_EcoRI_F<br>PPE68_1_HA_HindIII_R	GGGGGGAATTCATGCGATCCATGTCTTTTG<br>GGGGGGAAGCTTTCACGCGTAGTCCGGC	EcoRI<br>HindIII
pSMT3::PE31/PPE18 SWAP PPE68_HA	PPE68 P107_W144_Fw<br>PPE68 P107_W144_Rv<br>PPE18_M1_P106_Rv	ACGGTGCCCCCG CCCGAGATCGCCG<br>GGCGTCCTGGGCCCACATTCGGATGAAATA<br>GTC CGGCGATCTCGGGCGGGGGCACCGT	

	PPE18_A146_Fw	GACTATTTCATCCGAATGTGGGCCCAGGACGCC	
Δ*espG*$_1$	EspG$_1$ KO LF<br>EspG$_1$ KO LR<br>EspG$_1$ KO RF<br>EspG$_1$ KO RR	TTTTTTTTCCATAAATTGGTTCGAATCAGGCCGAATATG<br>TTTTTTTTCCATTTCTTGGATCAGCCAAAAATCTTGTC<br>TTTTTTTTCCATAGATTGGAGTCTGCTCGAACTACTTCC<br>TTTTTTTTCCATCTTTTGGTTGAGCCACGACACCAGATG	
pMV::*espG*$_1$*/espH/eccA*$_1$	Fw_PacI_EspG$_1$<br>Rv_ EccA$_1$_HindIII	GGGGGGTTAATTAAATGACCGGTCCGCTCG<br>GGGGGGAAGCTTTCACTCTCTCATATTGAGGTGTG	PacI<br>HindIII
pMV::*esxM-esxN-espG*$_5$	EsxMmar_XmnI_F<br>EspG$_5$mar_HindIII_R	GACTGAAAGAATTCCATATGACTGCACGCTTTATGACC<br>GACTAAGCTTTCAAACTCTGCTATGCGTTTTC	XmnI<br>HindIII
Quantitative PCR	SigA-Fw<br>SigA-Rv<br>esxA1_Fw<br>esxA1_Rv<br>esxA2 qPCR F<br>esxA2 qPCR R<br>esxA3 qPCR F<br>esxA3 qPCR R	GAAAAACCACCTGCTGGAAG<br>CGCGTAGGTGGAGAACTTGT<br>CACCAGCATTCATTCCCTTC<br>AGGTTCTGCAGCGAGTTGTT<br>ATTCGGCCTTCTGCTTGTTGG<br>TTCGGCCTTCTGCTTGTTGG<br>GCATCCAGCGCAATTCAGGG<br>GCGAGTTGTTGAGCTCCTGC	
